# Parahydrogen-induced polarization enables the single-scan NMR detection of a 236 kDa biopolymer at nanomolar concentrations

**DOI:** 10.1038/s41598-023-37202-0

**Published:** 2023-06-21

**Authors:** Franziska Theiss, Laura Wienands, Jonas Lins, Marcel Alcaraz-Janßen, Christina M. Thiele, Gerd Buntkowsky

**Affiliations:** 1 Eduard-Zintl-Institute for Inorganic and Physical Chemistry, Technical University of Darmstadt, Peter-Grünberg-Straße 8, 64287 Darmstadt, Germany; 2 Clemens-Schöpf-Institute for Organic Chemistry and Biochemistry, Technical University of Darmstadt, Peter-Grünberg-Straße 16, 64287 Darmstadt, Germany

**Keywords:** NMR spectroscopy, Chemical physics

## Abstract

Nuclear magnetic resonance (NMR) experiments utilizing parahydrogen-induced polarization (PHIP) were performed to elucidate the PHIP activity of the synthetic 236 kDa biopolymer poly-γ-(4-propargyloxy)-benzyl-L-glutamate) (PPOBLG). The homopolypeptide was successfully hyperpolarized and the enhanced signals were detected in 11.7 T solution NMR as a function of the PPOBLG concentration. The hydrogenation with parahydrogen caused signal enhancements of 800 and more for the vinyl protons of the side chain at low substrate concentration. As a result of this high enhancement factor, even at 13 nM of PPOBLG, a single scan ^1^H-NMR detection of the hyperpolarized protons was possible, owing to the combination of hyperpolarization and density of PHIP active sites.

## Introduction

Nuclear magnetic resonance (NMR) is one of the most commonly used and versatile analytical and diagnostic techniques in chemistry, medicine, biophysics and other areas of natural sciences. Nevertheless, NMR analysis has the crucial limitation of low sensitivity. A further inconvenience is signal broadening in the case of macromolecular systems (see e.g. ref.^1^).

To counter these limitations, hyperpolarization techniques are used to transfer spin polarization from a highly polarized spin reservoir to the nuclear spins and thus increase their signal/noise ratio. Major benefits of these techniques include reducing the time and amount of substance required to obtain high-resolution NMR spectra as well as spectral simplification in case selective hyperpolarization. Hyperpolarization techniques such as dynamic nuclear polarization (DNP)^2,3^, parahydrogen-induced polarization (PHIP)^4–8^ or signal amplification by reversible exchange (SABRE)^9^ are therefore becoming increasingly important. The methods mentioned differ in the source of the polarization that provides the signal amplification at the investigated nuclei^10^. In case of PHIP, the hyperpolarization is created by the catalytic addition of a parahydrogen molecule to an unsaturated bond of a molecule. The resulting NMR spectra, which are called PHIP pattern, depend on the experimental protocol. Two limiting types of experiments are distinguished in the literature: PASADENA^5^, in which the hydrogenation and detection is carried out inside the NMR magnet, and ALTADENA^11^, in which the hydrogenation takes place outside the NMR magnet but the detection takes place after transferring the sample into the NMR magnet.

A major advantage of PHIP compared to most other hyperpolarization techniques is that it can be implemented on any modern NMR spectrometer or MRI tomograph with only minor investments in hardware (see review^12^ and references therein). PHIP leads to NMR signal amplification, which can reach a factor of 2000 (with 11.7 T magnets) and more for protons as compared to thermal polarization. PHIP experiments were successfully applied in many different areas of research. For example, to study mechanistic problems in organic and inorganic synthesis^13–21^, or to monitor chemical processes in microreactors^22–24^. It has also been used for the hyperpolarization of amino acids and peptides^25,26^, and other biological metabolites^27^, such as pyruvate^28^, and fumarate^29–32^. In particular PHIP labelled peptides are potentially applicable as pharmacologically active biomarkers for studies of physiological processes, as shown recently in studies of the sun-flower trypsin inhibitor (SFTI-1)^33^, and the antiplatelet aggregation inhibitor eptifibatide^34^, a derivate of the disintegrin protein barbourin in the venom of certain rattlesnakes.

While most PHIP experiments were performed on small to medium sized molecules typically below 2 kDa, to the best of our knowledge there is until now only a single example of large molecules hyperpolarized with PHIP. In their seminal paper, Münnemann et al.^35^ successfully performed PHIP-experiments on two hyperbranched polymers with MWs of 31.5 kDa and 66 kDa. While this paper opened a new potential area of application for the PHIP hyperpolarization technique, this field was not further explored until now, most probably owing to the challenges of performing PHIP on large molecules. Typical problems, which can be encountered here, are line-broadening due to long rotational correlation times of the molecule, which leads to cancellation of the PHIP anti-phase signals or steric problems which hinder or prevent the binding of the catalyst to the unsaturated moiety, e.g., in random coil conformations, where the majority of reactive groups is hidden inside the polymer. While the first problem is intrinsic to solution NMR of any macro-molecule, the second problem can be circumvented by selecting substrates with ordered conformations where a major part of the unsaturated groups is on the outside of the molecule and accessible for a reaction. In particular, biopolymers, such as polypeptides with long helical segments and PHIP-active moieties in their side chains, could be an interesting new substrate class for PHIP hyperpolarization. In order to verify this hypothesis, we performed PHIP experiments on the 236 kDa polymer (poly-γ-(4-propargyloxy)-benzyl-L-glutamate)^36–38^ (PPOBLG, **1**). In the first step, we demonstrate that PPOBLG is a PHIP-active polymer. In the next step, we determine the minimum concentration, at which PHIP enhancement is still observable in a single scan by successive dilution. Finally we discuss, how these results can be combined with other techniques, such as ZULF-NMR^39^ in order to achieve new possibilities for the characterization of e.g. polymer-surfaces.

## Material and methods

### Nuclear magnetic resonance and PHIP–NMR

All NMR experiments were performed on a Bruker Avance III NMR spectrometer at an 11.7 T (500 MHz ^1^H frequency) OXFORD magnet in 5 mm NMR tubes. All PHIP experiments were performed under PASADENA conditions (hydrogenation with *p*-H_2_ inside the magnet). For the hydrogenation 95% *para*-enriched hydrogen was bubbled through the sample with a pressure of 7 bar at 25 °C. For the exact used *p*-H_2_ bubbling time see Table 1. After bubbling stopped the gas flow was immediately changed to a static helium overpressure. For time critical measurement (0.071 µM and lower) the measurement was started immediately after bubbling without gas changing. Subsequently, a single scan ^1^H-NMR spectrum of the hyperpolarized mixture was recorded, which in the following is called PHIP spectrum. In order to determine the enhancement factor by PHIP, a second proton spectrum is subsequently recorded, at a time point when the temporary polarization has completely decayed, which will be called thermal spectrum in the following. A flip angle of 90° and 16 scans are used for the recording of the thermal spectrum and a 45° flip angle and a single scan are used for the PHIP measurements. The spectra were processed using MestReLab Research MestReNova 14.2. All chemical shifts (δ) are reported in ppm relative to TMS (δ = 0.00). For detailed information see SI.

**Table 1:** Samples and optimized measurement condition.

Polymer concentration/µM	Amount of polymer/mmol	Catalyst concentration/mM	Bubbling time/s	^1^H Enhancement of the signal at 5.5–5.0 ppm (H_2a_)^1^
10.27	6.16·10^−6^	3	45	23 $\pm$ 1.3
5.13	3.08·10^−6^	3	45	33 $\pm$ 4.7
2.57	1.54·10^−6^	3	45	48 $\pm$ 6.6
1.03	6.16·10^−7^	3	45	63 $\pm$ 11
0.77	4.62·10^−7^	3	45	67 $\pm$ 8.1
0.66	4.00·10^−7^	3	45	83 $\pm$ 2.9
0.39	2.37·10^−7^	3	20	167 $\pm$ 7
0.15	9.49·10^−8^	3	20	217 $\pm$ 22
0.12	7.12·10^−8^	3	20	394 $\pm$ 48.8
0.071	4.24·10^−8^	1.54	10	~ 666^2^ $\pm 88$
0.053	3.18·10^−8^	1.54	10	~ 801^2^ $\pm 137$
0.035	2.12·10^−8^	0.77	5	/

^1^The error ranges were calculated by the mean of resulting enhancment factors, when choosing other baselines. ^2^The thermal signal for calculating the enhancement factors were determined by extrapolation from the higher concentrations.

### Synthesis of the polymer

The polymer poly-γ-(4-propargyloxy)-benzyl-L-glutamate^38^ is synthesized according to a literature procedure^36,38,40,41^ via ring-opening polymerization of the *N*-carboxyanhydride (NCA)^40^ of the respective functionalized amino acid using dimethylethanolamine (DMEA)^42^ as initiator (polymerization in DCM in the glovebox under inert atmosphere, M/I = 500)^42^. This results in a homopolymer (poly-γ-(4-propargyloxy)-benzyl-L-glutamate) (PPOBLG) with a number-average molecular weight (M_n_) of 2.36·10^5^ g/mol and a polydispersity index (PDI) of 1.25. The molecular weight distribution (MWD) was determined by gel permeation chromatography (GPC) relative to polystyrene standards (in THF with 0.1% tetrabutyl ammonium bromide, concentration: 1 mg/mL, 25 °C, column: PSS SDV analytical linear XL 5 µm). According to the literature, the peptide has an α-helical conformation, which was confirmed to be right-handed using circular dichroism (CD) spectroscopy (negative Cotton-effect at 220 nm). The assignment of the alkyne ether polymer **1** can be seen in the SI.

### Sample preparation

Chloroform-d and the catalyst [1,4-bis-(diphenylphosphino)-butane](1,5-cyclooctadiene)-rhodium(I) tetrafluoroborate ([Rh(dppb)(COD)]BF_4_) were purchased from Sigma-Aldrich and employed without further purification.

For the concentration-dependent ^1^H-NMR measurements, a known amount of PPOBLG **1** was dissolved in chloroform-d. At the same time, the rhodium catalyst [1,4-bis(diphenylphosphino)butane](1,5-cyclooctadiene)rhodium(I) tetrafluoroborate [Rh(dppb)(COD)]BF_4_ was dissolved in chloroform-d. Depending on the desired concentration, the two solutions were mixed in different ratios and, if necessary, topped up with chloroform to obtain a filling volume of 600 µL.

Table 1 shows the individual compositions of the concentration series as well as the obtained enhancement factors.

### Evaluation of the effective enhancement related to the thermal signal

Data were processed using MestreLab Research MestReNova 14.2. As the PHIP signal contains both positive and negative signal components, the enhancement factor (*ε*), which represents the ratio of the signal intensity of a PHIP signal, was determined by first correcting the phase, followed by calculating the absolute value of the real part (i.e. deleting the imaginary part) of the spectrum and integrating over the line. These integrals were compared to the corresponding integrals of the thermal signal after weighing with the individual measurement parameters, such as the number of scans (*NS*) and the receiver gain (*RG*). The calculation is performed according to Eq. (1).

1 $$\varepsilon = \frac{{Int}_{PHIP}/{N}_{PHIP}}{{Int}_{Thermal}/{N}_{Thermal}}$$

With the normalization factor *N*_j_ = (*RG*_j_)*/*(*NS*_j_)*/*(*number of proton*_*j*_) of every state j.

The value obtained corresponds to an effective enhancement of the PHIP spectrum in relation to the thermal spectrum. Since we are interested in the first partial hydrogenation from alkyne polymer **1** to allyl polymer **2**, the enhancement factor of the allyl ether signal was calculated. The integration range was 5.4–5.1 ppm for the thermal signals and 5.5–5.0 ppm for the PHIP signals in order to accommodate for the slightly larger line-widths of the PHIP signals. Possible contributions to the integral from neighboring signals that overlap with the hyperpolarized signals are negligible due to their low intensity.

## Results and discussion

PPOBLG **1** is a homopolypeptide consisting of approximately 810 repetition units of γ-(4-propargyloxy)-benzyl-L-glutamate. The glutamate derivative contains a propargyl ether unit, which, owing to the triple bond, is known to be a very efficient PHIP label^32^ and can be hydrogenated twice, first to the allyl- then to the alkyl-ether.

**Figure 1:**
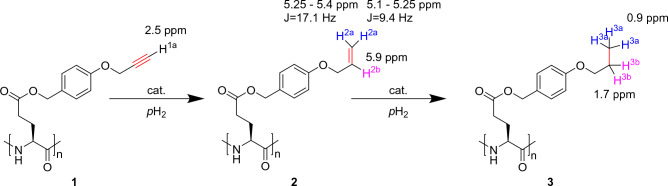
Hydrogenation reaction of PPOBLG to the allyl ether polymer **2** and the alkyl ether polymer **3**.

We performed hydrogenation studies with **1.** The hydrogenation reaction of the side chain of the polymer proceeds as shown in Fig. 1. A hydrogenation catalyst is added to polymer **1** and subsequently the mixture is flooded with thermal hydrogen and parahydrogen gas, respectively. The first hydrogenation product formed is the allyl ether polymer **2**. Subsequently, complete hydrogenation can occur, forming the alkyl ether polymer **3**. A complete assignment of the NMR signals of the three substances is given in the SI.

**Figure 2:**
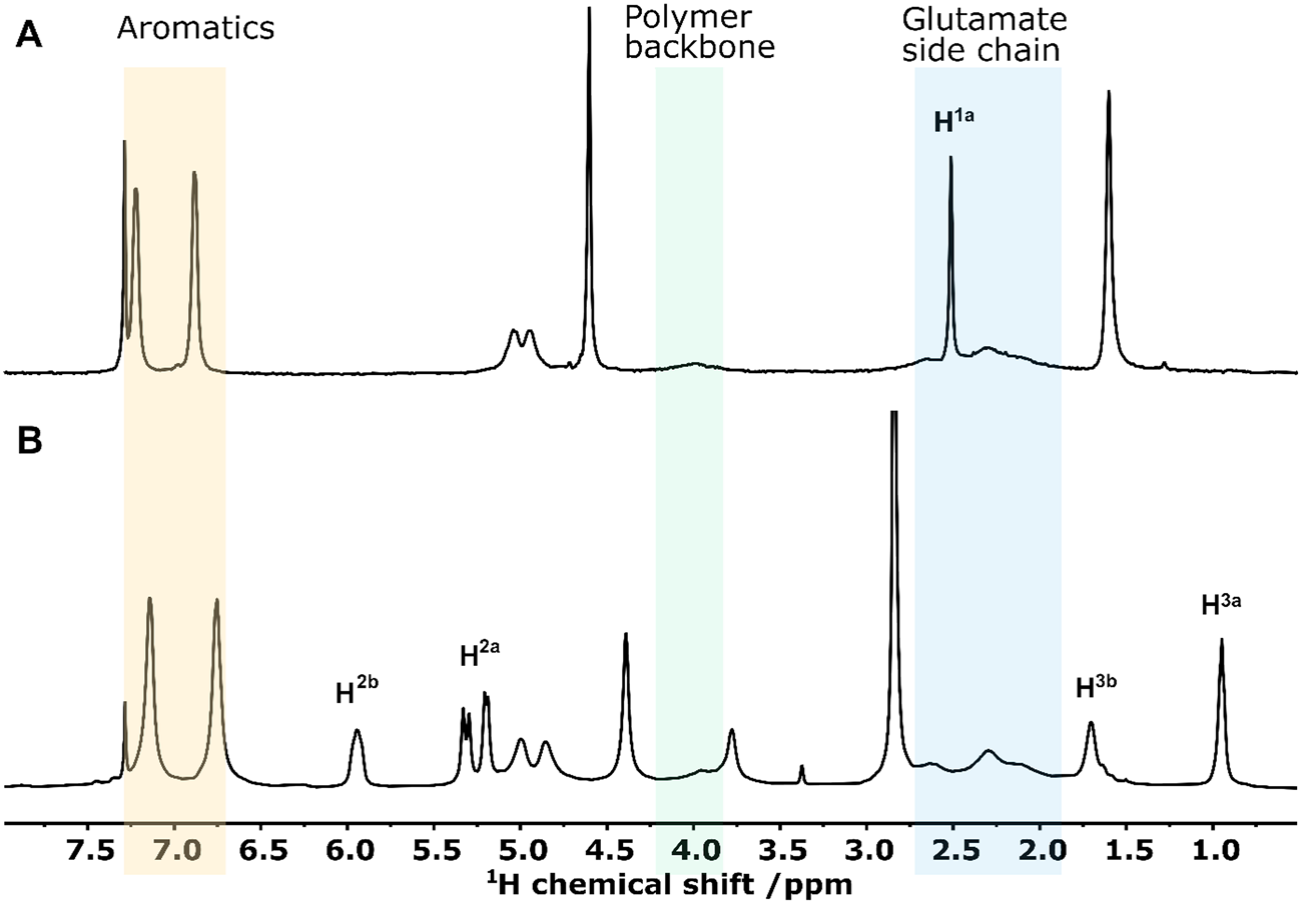
^1^H-NMR spectrum of (**A**): pure 16 mg PPOBLG **1** in CDCl_3_ and (**B**): the spectra after hydrogenation using a hydrogen feed of 7 bar thermal hydrogen for about 20 min at 25 °C. (To obtain the product spectrum B without catalyst residues, the sample was precipitated and redissolved.) The newly formed reaction products **2** and **3** can both be seen in (**B**). Each spectrum is measured in a single scan. In order to avoid too much crowding of the figure, the full assignment and further notes are given in the SI.

In a first step we tested the hydrogenation activity of the polymer with thermal hydrogen. Figure 2 compares the ^1^H-NMR spectrum of the reactant **1** and the spectrum of the products after hydrogenation.

Figure 2 shows the general change from triple bond signals (H^1a^; 2.5 ppm) to the newly formed allyl ether polymer **2** and the alkyl ether polymer** 3**. The low-field singlet at 5.9 ppm is assigned to the newly bonded single proton of the double bond (**2**). The signals at 5.1–5.4 ppm belong to the two geminal protons of allyl ether polymer **2**. Interestingly, the terminal vinylic protons can even be assigned based on the multiplicity observed despite the presence of broad lines typical of a polymer sample. The larger coupling constant with 17.1 Hz (doublet 5.25–5.4 ppm) can be related to the H^2a^ which is trans to H^2b^. Consequently, the smaller coupling 9.4 Hz is related to the H^2a^ which is cis to H^2b^)^6^. The intense high-field singlet at 0.9 ppm (H^3a^) is assigned to the terminal methyl group of the alkyl moiety of the doubly hydrogenated product **3**. The newly formed methylene group is visible in the NMR spectrum (B) as a singlet at 1.7 ppm. As both product species **2** and **3** are visible in the spectrum, a full conversion of PPOBLG (16 mg in this experiment) to **3** by hydrogenation can be excluded after the hydrogenation time of 20 min at 7 bar. From the deconvolution of the spectrum the ratio of molecules **2**:**3** is estimated to be 2:1. The important signal of the alkyne ether polymer **1** at 2.5 ppm can no longer be distinguished from the broad signal of the glutamate side chain at 1.9–2.7 ppm. The reaction progress was observed experimentally by PHIP (see SI).

### Hydrogenation with parahydrogen—PHIP activity

**Figure 3:**
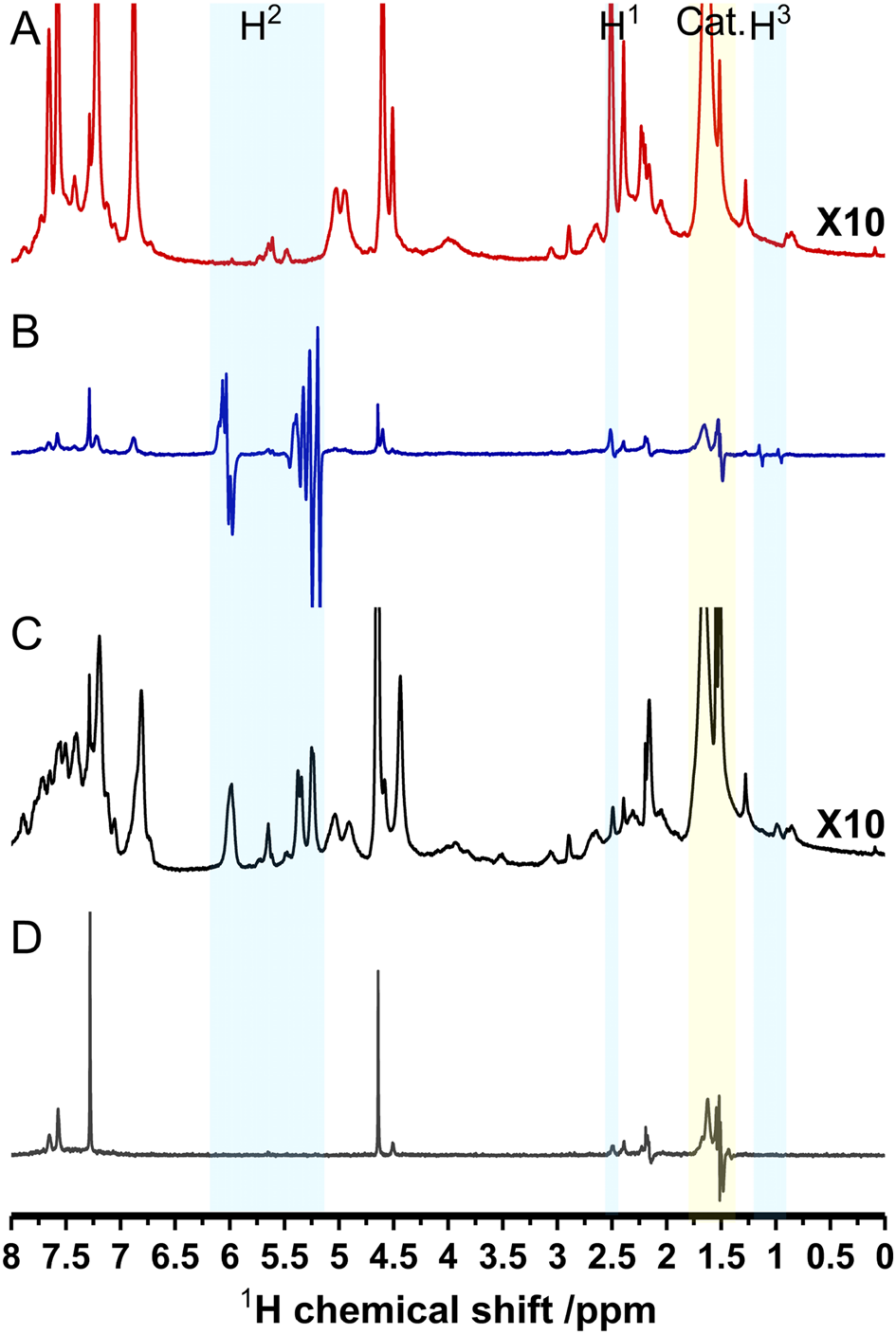
^1^H-NMR spectra of (**A**): the reaction mixture (16 scans) before hydrogenation, (**B**): the PHIP spectrum after 45 s of 7 bar *p*-H_2_ bubbling, 1 scan, (**C**): the thermal spectrum (16 scans) and (**D**): spectrum of the neat catalyst in the presence of parahydrogen. All measured in CDCl_3_.

After the assignment of the important signals of the products **2** and **3** and reactant **1** (for details see SI), the PHIP activity can now be investigated. For this purpose, a concentration of 6.16·10^−6^ mmol of PPOBLG **1** is used to facilitate the observation and the assignment of the signals. Figure 3 compares the ^1^H-NMR spectrum of the PPOBLG **1** (10.3 µM) before hydrogenation (A), with its PHIP spectrum (B) measured directly after the hydrogenation (45 s of pH_2_ bubbling at 7 bar) and the resulting thermal spectrum (C).

The PHIP spectrum (Fig. 3B) shows a large number of strong signals with the characteristic PHIP signature, resulting from the superposition of positive and negative signals components. From this it can be concluded that the label successfully produces parahydrogen-induced polarization. By comparison with the spectra in Fig. 2, the PHIP signals at 5.1–5.4 ppm can be assigned to the geminal (terminal) protons of the vinyl group (polymer **2**) and the PHIP signal at 5.9 ppm to the internal (single) proton of the vinyl group. As the PHIP signal at 1.1 ppm is not accompanied by a visible signal in the thermal spectrum or in the spectrum of the pure catalyst, we attribute this signal to a low concentrated and most probably transient complex of the substrate coordinated to the catalyst, whose concentration is too weak to be detectable without hyperpolarization, i.e., in thermal spectra. While the weak PHIP signal at 0.9 ppm, indicates the onset of the second hydrogenation to **3**, the low intensity of this signal in the thermal spectrum shows, that only a very minor fraction of the molecules is fully hydrogenated after a bubbling time of 45 s. Therefore, it can be concluded, that at low concentrations and short exposure times, a selective hydrogenation to the allyl ether polymer **2** occurs. By integrating the signals of the vinyl moiety in comparison to the integral of the alkyne signals **1**, the reaction conversion for the first hydrogenation step is determined to be about 73%, corresponding to an approximate conversion of 4.5·10^−6^ mmol. The calculated effective enhancement factor is 23.

In order to assign the remaining hyperpolarized signals in B, the PHIP spectrum is compared to the spectrum of the neat catalyst [Rh(dppb)(COD)]BF_4_ interacting with *p*-H_2_ (Fig. 3D). From this comparison it is evident, that the signal at 1.5 ppm can be assigned to a complex of *p*-H_2_ and the catalyst. Finally, the remaining signal in spectrum D at 4.6 ppm is attributed to dissolved hydrogen gas. Detailed spectra of the employed rhodium catalyst ([Rh(dppb)(COD)]BF_4_) before, during and after interactions with hydrogen as well as a comparison with the PPOBLG **1** and the reaction mixture are given in the SI (Fig. S3 and S4).

### Influence of the substrate concentration on the enhancement factor

In the next step the influence of the polymer concentration on the signal enhancement factor is investigated. In these experiments the duration of the hydrogen bubbling and the catalyst concentration remain constant, and the substrate concentration is varied.

**Figure 4:**
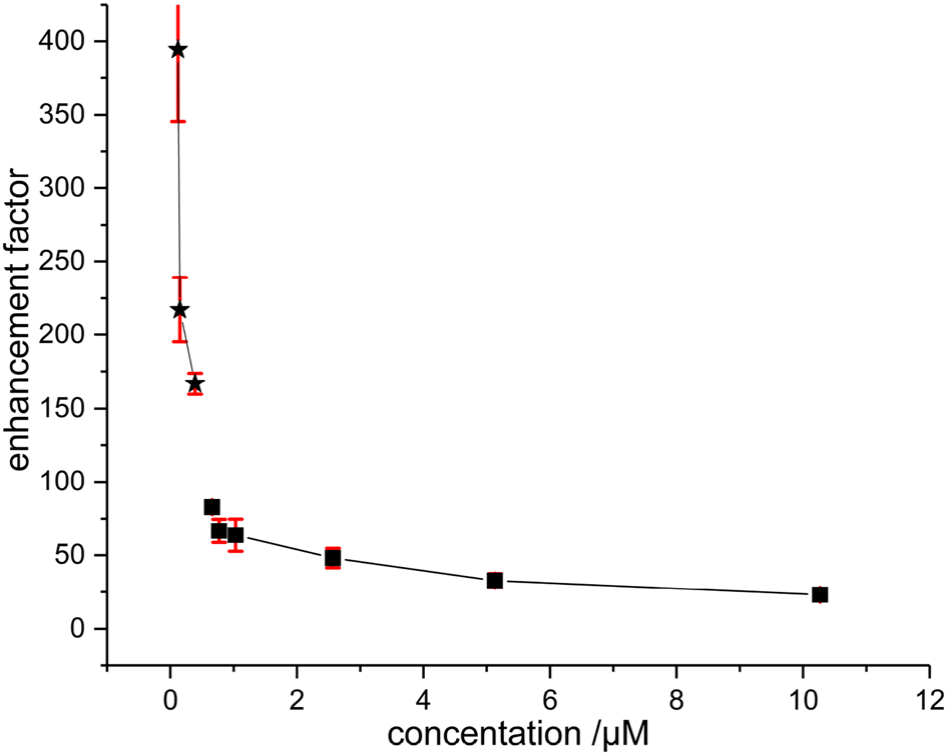
Dependence of the enhancement factors on the substrate concentration (Table 1). The measurements of 10 µM down to 0.5 µM were performed using 3 mM cat. and 45 s bubbling of 7 bar *p*-H_2_. The concentrations between 0.5 and 0.12 µM (stars) were measured with 3 mM cat. and 20 s of 7 bar *p*-H_2_. All PHIP spectra were measured in a single scan. Note 10.27 µM is not shown here.

Figure 4 shows the enhancement factor determined as a function of the substrate concentration (see Table 1). It is evident that the enhancement factor grows upon lowering the concentration. This is explained by the fact that at high concentrations the relative amount of hydrogenated substrate molecules is only a minor fraction of the overall number of substrate molecules, which is the denominator of Eq. (1). At high concentrations, the enhancement factor is mainly influenced by the turnover per time, which depends significantly on the catalyst or parahydrogen concentration, which are both held constant in the experiment with 1 µM up to 10 µM. At low concentrations (0.12–0.39 µM), where the size of the thermal signal of the allyl ether polymer **2** is no longer large compared to the noise level, the determined value of the enhancement factor depends significantly on the baseline chosen, as shown by the error bars (Fig. 4).

As the assignment of the terminal vinylic protons is possible, another observation can be made in these spectra: the signals of the two H-atoms adding to the double bond on the same face (syn-selectivity, H2b and H2a-cis) exhibit a stronger enhancement as compared to the proton H2a-trans^6^.

**Figure 5:**
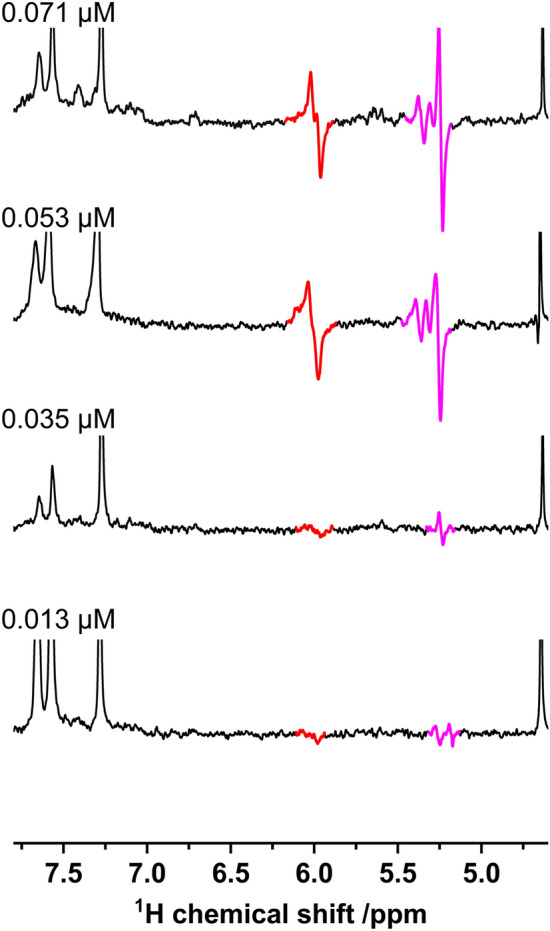
Illustration of the relevant PHIP signals of allyl ether polymer **2** at low concentrations. The samples with 71 nM and 52 nM of substrate are measured with 1.5 mM catalyst, 10 s of bubbling at 7 bar and 1 scan. At 35 nM of substrate 0.7 mM of catalyst and 5 s of bubbling are employed. Finally, the 13 nM of substrate are measured with 1 scan, 1.5 mM catalyst and 6.5 s of 7 bar bubbling *p*-H_2_.

The detection limit for the product-specific signals of the allyl ether polymer **2** in the thermal spectrum has already been reached at 71 nM. Even accumulating 128 scans could not improve the signal to noise ratio of the thermal signal in a reasonable time to permit a calculation of the enhancement factor. Using hyperpolarization allows the observation of the reaction even at these very low concentrations. The question arises, for how long we can detect PHIP before the resolution and the detection limit is reached (Fig. 5). In order to be able to record the PHIP signature at very low concentrations, we optimized the bubbling time and the catalyst concentration for each measurement. At this point it should be mentioned that it is important to select an optimum hydrogenation time. At too short hydrogenation times, the turnover is not high enough to generate a substantial amount of polarization and at too long hydrogenation times, T_1_-relaxation effects reduce the amount of created polarization. Since there is no allyl signal left in the thermal spectra, we cannot say anything about the exact yield. We thus assume the reaction to go to completion. As the concentration was too low to record a thermal spectrum even with more than 512 scans, the sensitivity limit was reached. To be able to give an approximate estimation of an enhancement factors we do an extrapolation of the thermal allyl signals at the higher concentrations, resulting in Ɛ = 666 at 0.071 µM and Ɛ = 801 at 0.053 µM concentration (see SI for details). At even lower concentrations, the extrapolation was no longer applicable, as the reaction parameters differ too much from the data used for the extrapolation.

**Figure 6:**
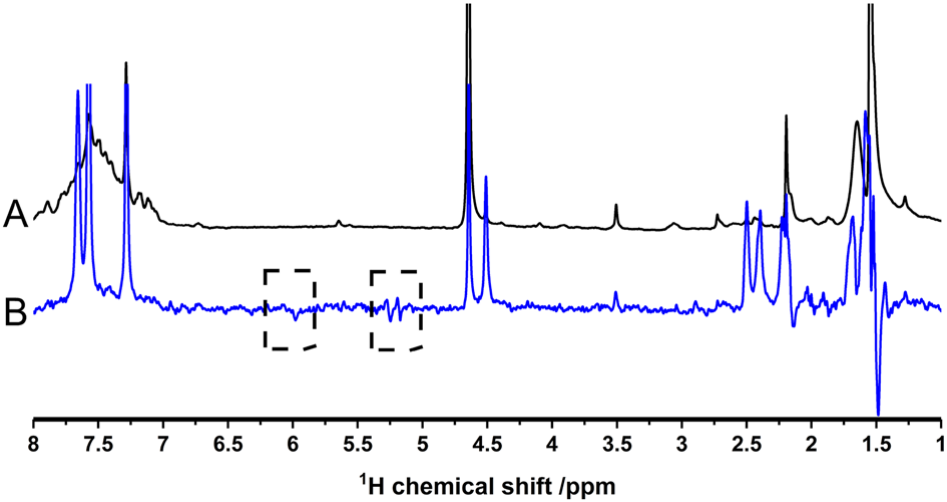
Comparison of (**A**): the thermal spectrum acquired with 16 scans and (**B**): the PHIP spectrum of a 13 nM polymer solution. The measurement conditions are: 13 nM PPOBLG, 1.5 mM [Rh(dppb)(COD)]BF_4_ with 6.5 s of 7 bar *p*-H_2_ feed. The PHIP spectrum shows a clear PHIP signal of the terminal protons of the vinyl group at a single scan. Note: the PHIP signal at 1.5 ppm is from the catalyst.

Figure 6 displays the spectra of the lowest concentration (0.013 µM) of PPOBLG **1**, where, after hydrogenation, a PHIP signal of the geminal protons of the vinyl group of the allyl ether polymer **2** at 5.1–5.4 ppm was still observable in a single scan experiment. As we could not record the thermal reference signal at those low concentrations (0.013 µM), we can only give a lower boundary for the enhancement factor of Ɛ = 801, which is the value obtained for the concentration of 0.053 µM where an enhancement factor could be determined by extrapolation. In addition, there is also a very weak PHIP signature of the internal vinyl proton visible at 6 ppm. These observations point at the possibility of detecting the signal of the polymer via hyperpolarization at concentrations at which no thermal signal can be observed anymore.

A particular challenge in the PHIP hyperpolarization of the PPOBLG is the, compared to smaller oligopeptides, relatively short T_1_ relaxation time of the hyperpolarized protons. At 500 MHz typical T_1_ values of the smaller oligopeptides are above 3 s. In contrast the T_1_ for the hyperpolarized protons next to the ether bond of the PPOBLG **1** (4.6 ppm s, 2H, –ArOCH_2_–) is only about 1.1 s, i.e., a factor of three smaller. This shortens the observation window of the hyperpolarization since relaxation processes are playing a dominant role in the polarization decay. For technical reasons it is necessary to put a waiting delay of 1.5 s between end of hydrogenation reaction and NMR detection, mainly in order to give the sample time to restore its homogeneity after the bubbling and to account for internal switching times. The problem is that during this delay already a fraction of the polarization is destroyed by relaxation processes. They are of particular importance at very low substrate concentrations, where a substantial part or all of the substrate is consumed after a short time, leading to a decreasing rate of production of hyperpolarized molecules and signal loss already during the hydrogenation time, which is further increased during the delay time. At higher substrate concentrations (0.39 µM up to 10 µM) this is a lesser problem as the continues hydrogenation process provides polarized substrate during the hydrogenation time at a practically constant rate. Owing to this concentration dependence of the impact of relaxation effects it is possible that the enhancement factors at low concentrations (in particular 13 nM) are underestimated.

Without the automatic PASADENA measurements, the reaction tracking at the very low concentrations down to 13 mM would not be possible. We wish to note at this point, that the present paper is mainly a proof-of-principle paper, i.e., we did not do a full optimization of all reaction parameters, such as temperature, reactants, pressure, in order to achieve the highest possible polarization, similar to our recent paper on fumarate hyperpolarization^31^.

## Summary and conclusion

For the first time successful PHIP hyperpolarization of a polypeptide with an MW above 100 kDa was demonstrated, employing the 236 kDa long chain homopolypeptide PPOBLG as example. PPOBLG exhibits very good PHIP activity even at nanomolar concentrations under PASADENA conditions. All PHIP signals of the reactants and hyperpolarized products could be attributed. The NMR spectra of the hydrogenation product only showed changes caused by the hydrogenation of the unsaturated moieties in the side chains but no changes in the polymer backbone. Contrary to our initial expectations of having difficulties due to line broadening caused by long rotational correlation times of the molecule that could lead to cancellation of the PHIP antiphase signals, the bigger experimental challenge were the relatively short T_1_ relaxation times of the polymer, compared to smaller oligopeptides in our previous studies^43,44^. In order to cope with those, it was necessary to use faster reaction conditions.

Employing these optimized reaction conditions (hydrogenation time, catalyst concentration and substrate concentration) and timing protocols of the NMR detection, a single scan detection of the signal of the PPOBLG at a concentration of 13 nM was possible. The signal-to-noise ratio and the obtained enhancement factor (> 800) in a 500 MHz spectrometer were significantly better than originally anticipated by us.

Our research shows that hyperpolarization by PHIP is not limited to small molecules or short oligopeptides but generally applicable to larger (bio)molecules with mw in the range of hundreds of kDa. The successful hyperpolarization of this long-chain polymer will hopefully motivate many researchers to perform hyperpolarization experiments on other long-chain polymers such as proteins and oligopeptides. There, the hydrogenation reaction and its kinetics can be followed or the structure, structure parts and products of this reaction can be analyzed by using small amounts of analyte. The ability to distinguish between the cis and trans configuration in the label can be interesting for polymer brushes. Especially with respect to isomeric different NOE effects. With regard to more complex 2D-NMR analyses an important step for practical applications is the possibility to transfer the created proton hyperpolarization to heteronuclei, such as ^13^C or ^15^N. With the help of ZULF-NMR^39^ this transfer is yet possible for small molecules. But for our example of a larger Polymer this problem is currently explored in our lab and the results will be published in a subsequent publication.

## Supplementary Information

Supplementary Information.

## Data Availability

All data generated or analyzed during this study are included in this published article and its Supplementary Information files.
