# Supplementary material for: Parahydrogen-induced polarization enables the single-scan NMR detection of a 236 kDa biopolymer at nanomolar concentrations
Source: Sci Rep. 2023 Jun 21;13:10117. doi: 10.1038/s41598-023-37202-0 (PMC10284874; doi:10.1038/s41598-023-37202-0)
Supplement: Supplementary file 1 — Supplementary Information. [file 41598_2023_37202_MOESM1_ESM.pdf]

Parahydrogen induced polarization enables the single-scan NMR detection of a 236 kDa biopolymer at nanomolar concentrations

*Franziska Theiss[a], Laura Wienands[a], Jonas Lins[a], Marcel Alcaraz-Janßen[b], Christina M. Thiele[b] and Gerd Buntkowsky[a]**

Table of Contents

1. General 2
2. NMR experiments 2
3. NMR of PPOBLG 4
4. Precipitation 5
5. NMR of both produkts 2 and 3 5
6. Information about the catalyst 7
7. Reaction progress during hydrogenation 10
8. Impurities 11

Experimental Methods

1. General

Chemicals

Chemicals used for the synthesis were purchased from the manufacturers or chemical distributors Sigma-Aldrich, Honeywell, Carl Roth, Fisher Scientific and TCI. The extra dry solvents used were taken from septa bottles, stored over molecular sieve. The *n*-hexane used in the NCA synthesis and purification, was dried over sodium and benzophenone under argon and then distilled.

Chloroform-d, methanol and the catalyst [1,4-bis-(diphenylphosphino)-butane](1,5-cyclooctadiene)-rhodium(I) tetrafluoroborate ([Rh(dppb)(COD)]BF_4) were purchased from Sigma-Aldrich.

2. NMR experiments

Nuclear Magnetic Resonance and PHIP-NMR

All NMR experiments were performed on a Bruker Avance III NMR spectrometer at an 11.7 T (500 MHz ^{1}H frequency) OXFORD magnet in 5 mm 528-TR-7 NMR tubes from Rototec Spintec. All PHIP experiments were performed under PASADENA[1] conditions (hyperpolarization of p-H_2 inside the magnet). To generate the PHIP signal, parahydrogen was guided through 1/16 inch Teflon tubing (1.6 mm [outer diameter], 0.5 mm [inner diameter]) connected to a glass capillary (0.36 mm [outer diameter], 0.15 mm [inner diameter]), centered in the NMR tube. The end of the capillary was close to the bottom of the tube and below the position of the detection coil so that the gas flow passes through as much of the sample as possible. For more information about the automatized PHIP-setup and the measurement conditions, see our recent publication.[2] The parahydrogen (p-H_2) enrichment was performed with a parahydrogen generator from Advanced Research Systems Inc. comprising a DE204A cryostat and an ARS-4HW compressor. The cryostat is cooled to 30 K and delivers >95 % para enriched hydrogen. For the hydrogenation this parahydrogen was bubbled through the sample with a pressure of 7 bar at 25 °C. After bubbling stopped the gas flow was immediately changed to a static helium overpressure (7 bar). For time critical measurement (0,071 µ and lower) the measurement was started immediately after bubbling without gas changing. Afterwards a single scan ^{1}H-NMR spectrum was recorded. This momentary image represents the so-called PHIP spectrum. For comparison of the enhancement, another proton spectrum is subsequently recorded, at a time point when the temporary polarization has completely decayed (thermally relaxed spectrum). All normal NMR experiments were acquired with pulse

sequences from the Bruker pulse sequence library. The PHIP spectra were acquired with customized pulse sequences adjusted to the PHIP setup.[2]

All chemical shifts (δ) are reported in ppm relative to TMS (δ = 0.00). In order to always achieve the maximum signal, a flip angle of 90° and 16 scans are used for the normal proton measurements. 45° flip angle and a single scan are used for the PHIP measurements. 45° is the standard excitation pulse in PASADENA PHIP for direct detection of two-spin antiphase nuclear spin order.[1]

Processing of the Spectra

The spectra a processed using MestreLab Research MestReNova 14.2. If not described otherwise, the spectrum was processed as indicated in Table 1

Table 1: detailed process parameters of the normal/ thermally relaxed spectrum and the PHIP spectrum.

	Normal Protons	PHIP
Size of FID	15k	32k
Zerofilling to spectrum size	64k	64k
Acquisition time	2.9 s	6.5 s
Spectral width	10 ppm	10 ppm
Line broadening	5 Hz	1 Hz**
Phase correction	manually	manually
Baseline correction	bernstein polynomial fit* 3rd order	bernstein polynomial fit 3rd order
Receiver gain	203	4
Numbers of Scans	16 (d1=10s between scans)	1
Integration ranges	5.4-5.1 ppm	5.5-5.0 ppm***

*At concentrations below 0.66 µM a multiple point baseline correction was carried out.

** As the PHIP signals are dispersive the line broadening was reduced to minimize signal cancellation.

*** At concentrations below 0.15 µM it was difficult to calculate the integral as the sum of the modulus of the respective PHIP signal due to the poor signal to noise ratio, the integrals obtained are more error loaded.

3. NMR of PPOBLG

No PHIP studies of the respective monomers could be performed for the following reasons: The N-carboxy anhydride (NCA), which is converted into the polymer by ring-opening polymerization, cannot be used for testing the PHIP activity due to its instability in air. The corresponding amino acid ester, which is converted into the NCA, could not be tested because it was not compatible with the selected measurement system, due to insolubility in the selected solvent. Thus only the polymer was investigated and the results will be described herein.

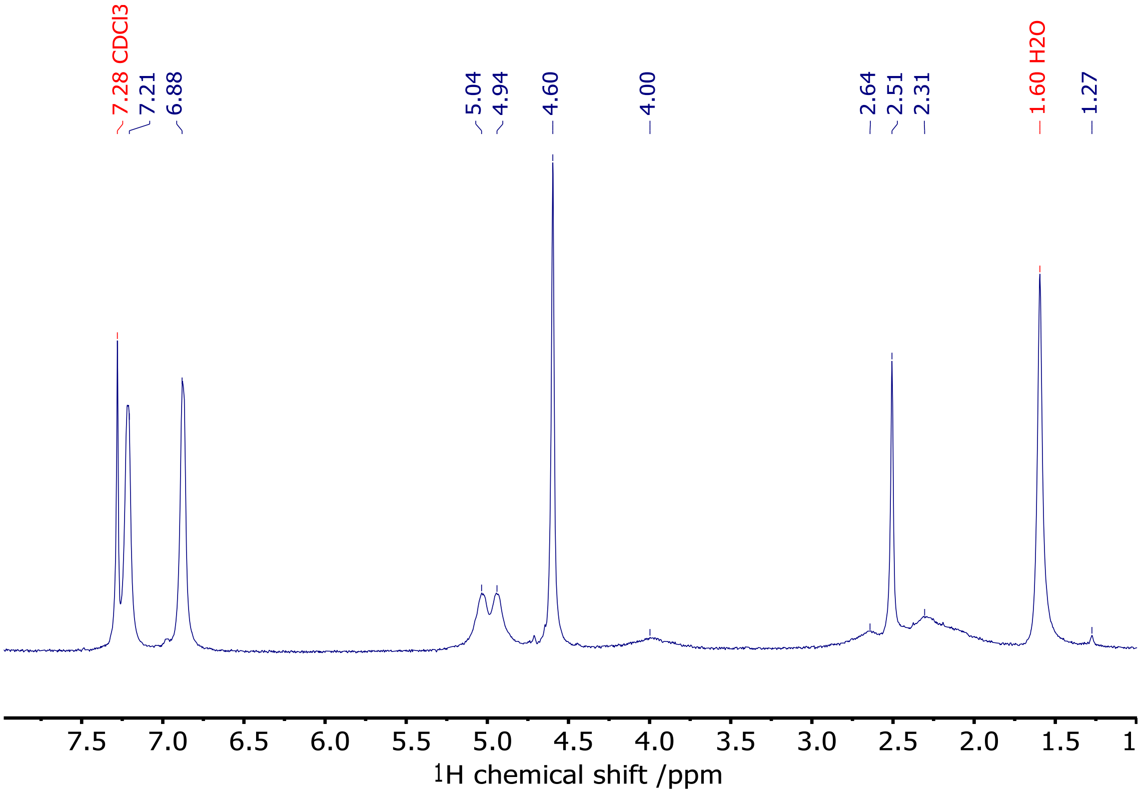

Figure 1: ^{1}H NMR spectrum of pure PPOBLG.

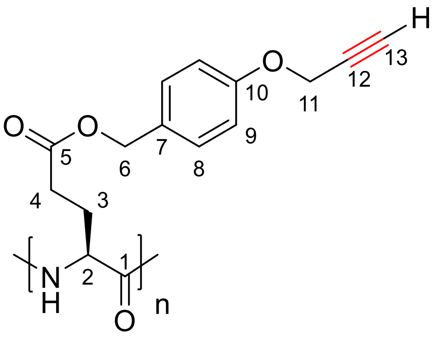

1

Assignment alkyne ether polymer **1**

^{1}H-NMR (500 MHz, 298.15 K, $CDCl_3$): δ = 1.91-2.65 (b, 4H, 3-H, 4-H), 2.51 (s, 1H, 13-H), 3.7-4.1 (b, 1H, 2-H), 4.60 (s, 2H, 11-H), 4.95-5.04 (2*b, 2H, 6-H), 6.89 (s, 2H, 9-H), 7.21 (s, 2H, 8-H) ppm.

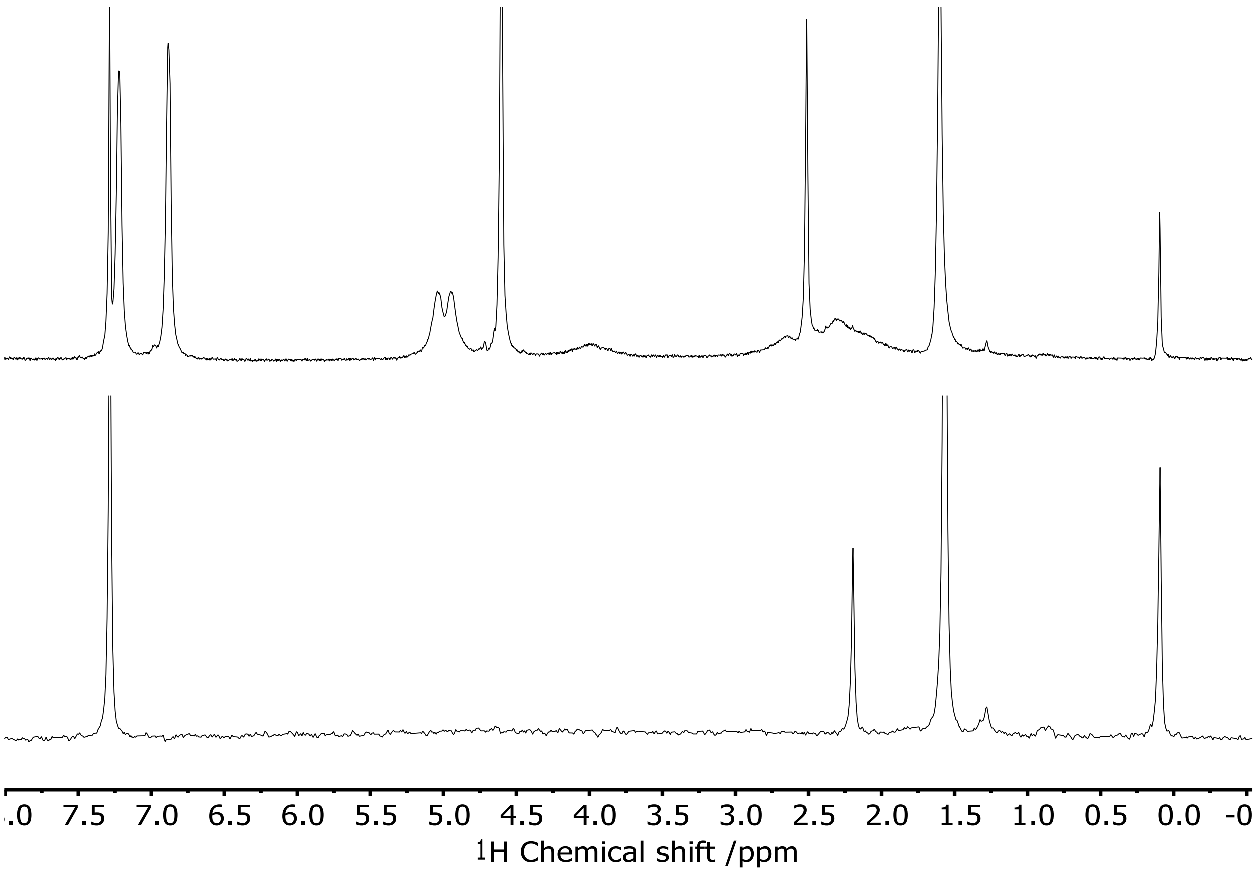

Figure 2: ^{1}H NMR spectrum of the pure PPOBLG 1 (above), as well as the pure spectrum of the used chloroform (below) to verify the source of the impurity visible in the spectra.

4. Precipitation

To obtain a product spectrum without catalyst residues, e.g. main paper figure 1B, the sample must be precipitated. For the precipitation experiment a mixture of 16 mg of the PPOBLG and 2.8 mg [Rh(dppb)(COD)]BF_4 in 1 mL $CDCl_3$ was used. Hydrogen was bubbled through the solution at 7 bar for 20 min. The resulting product can be precipitated out of the catalysator-containing reaction mixture by adding the reaction mixture to methanol and washing the precipitate twice with small amounts of cold water. Afterwards, the precipitated polymer can be dissolved in chloroform-d again for NMR analysis.

In addition to the two products, the spectrum obtained also contains impurities which were introduced in the course of precipitation, drying and redissolution. The signal at 2.88 ppm may be assigned to water in acetone which is typical for a freshly rinsed but not yet completely dried NMR tube. However, the impurity has no influence on further measurements and comparability.

5. NMR of both products 2 and 3

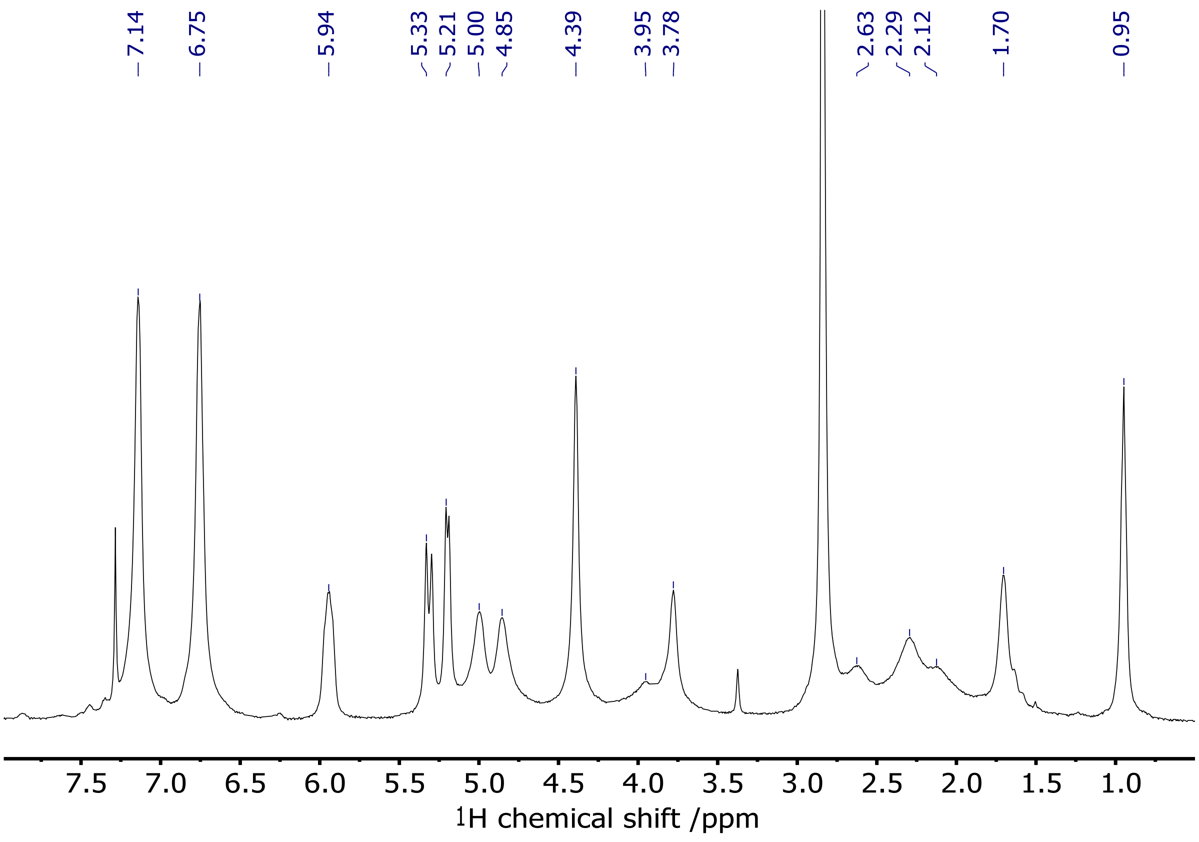

Figure 3: Single Scan ^1H NMR spectrum of 16 mg PPOBLG after a hydrogen feed of 7 bar thermal hydrogen for about 20 min at 25°C. The hydrogenated products 2 and 3 can both be seen.

Assignment allyl ether polymer **2**

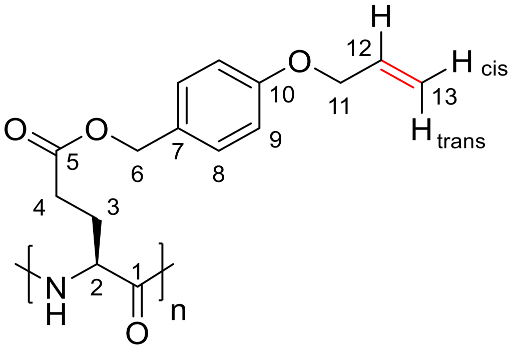

2

^{1}H-NMR (500 MHz, 298.15 K, $CDCl_3$):δ= 1.91-2.65 (b, 4H, 3-H, 4-H), 3.95 (b, 1H, 2-H), 4.39 (s, 2H, 11-H), 4.85-5.0 (2*b, 2H, 6-H), 5.1-5.4 (2*d, 2H, J_{trans}=17.1 Hz, J_{cis}=9.4 Hz, 13-H), 5.9 (m, 1H, 12-H), 6.76 (s, 2H, 9-H), 7.14 (s, 2H, 8-H) ppm.

Assignment alkyl ether polymer **3**

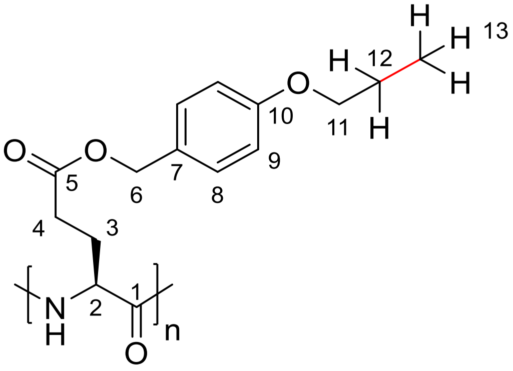

3

^{1}H-NMR (500 MHz, 298.15 K, $CDCl_3$): δ = 0.95 (s, 3H, 13-H), 1.7 (s, 2H, 12-H), 2.12-2.65 (b, 4H, 3-H, 4-H), 3.78 (s, 2H 11-H), 3.95 (b, 1H, 2-H), 4.85-5.0 (2*b, 2H, 6-H), 6.76 (b, 2H, 9-H), 7.14 (b, 2H, 8-H) ppm.

6. Information about the catalyst

The catalyst is commercially available with the CAS number: 79255-71-3.

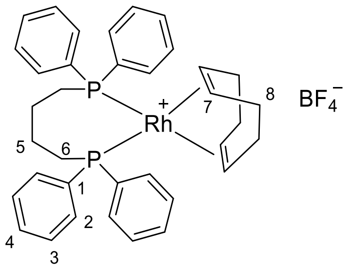

^{1}H-NMR (500 MHz, 298.15 K, $CDCl_3$): δ = 1.62 (s, 4H, 5-H), 2.19 (s, 4H, 6-H), 2.39-2.49 (b, 8H, 8-H), 4.51 (s, 4H, 7-H), 7.57-7.65 (m, 20H, 2-4H) ppm. Each atom was numbered only once, since both building blocks of the catalyst are mirror symmetric

The catalyst complex is activated by hydrogenation. The COD ligand is detached which slightly changes the structure of the complex and its resulting NMR spectrum.

Assignment after reaction:

^{1}H-NMR (500 MHz, 298.15 K, $CDCl_3$): δ = 1.52-1.54 (free cyclooctane), 1.63 (s, 4H, 5-H), 2.19 (s, 4H, 6-H), 7.11-7.89 (aromatic H atoms) ppm. The signal at 4.6 ppm is dissolved ortho-hydrogen.

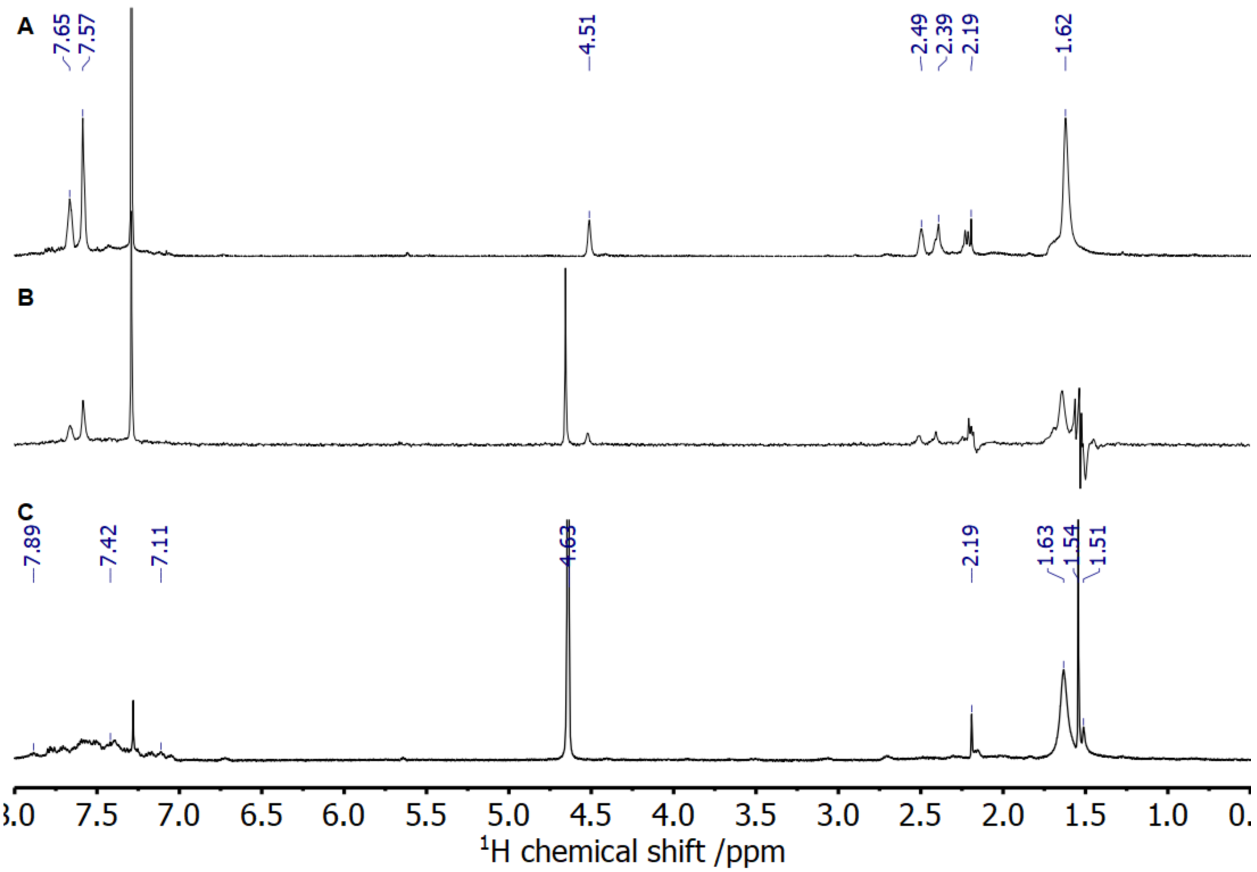

Figure 4: Comparison of spectra of A: the pure catalyst (in $CDCl_3$) with B: the spectrum which is obtained after para hydrogen has been passed through the dissolved catalyst for 15s and C: The thermally relaxed catalyst after a total of 50 s of hydrogen feed.

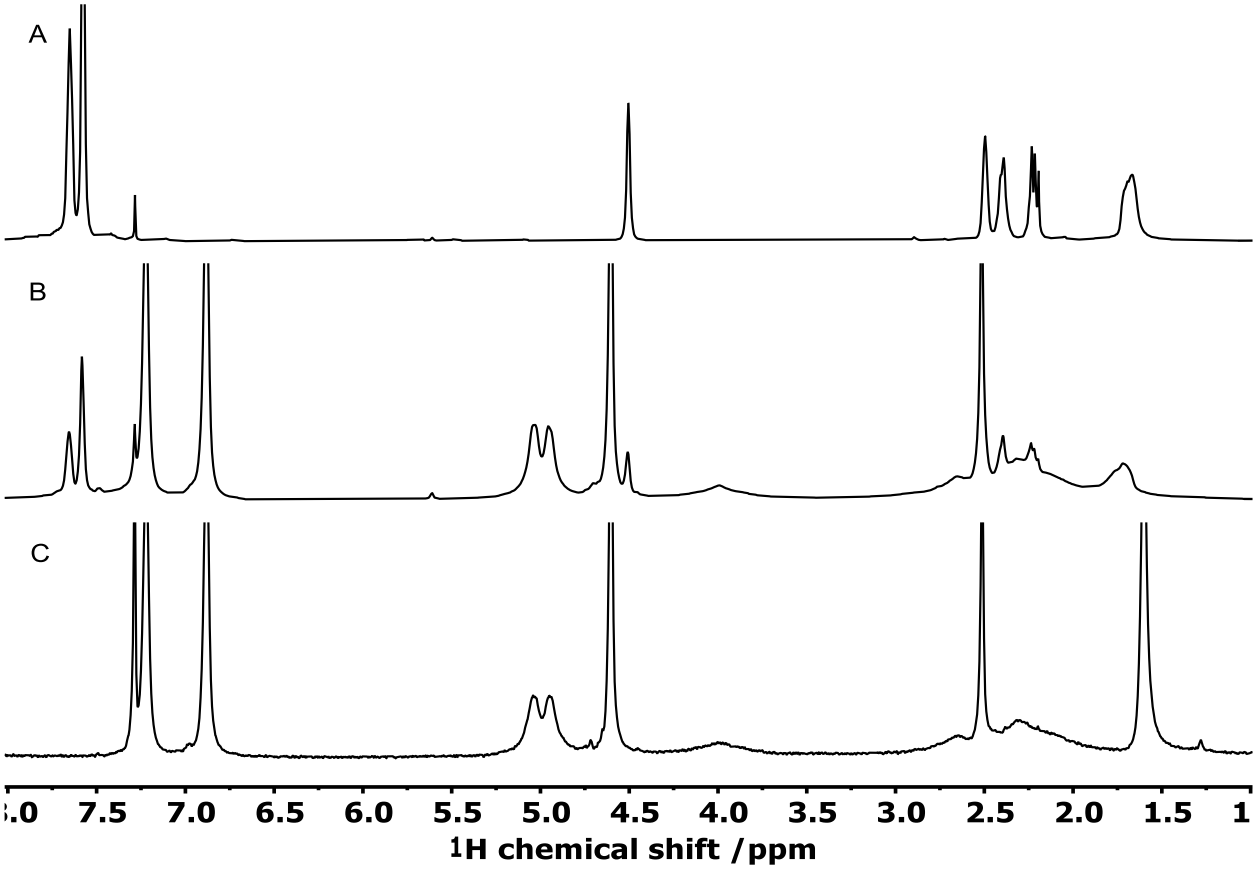

Figure 5: ^{1}H NMR spectrum of the pure catalyst in chloroform-d at 25°C (A) in comparison to the reaction mixture containing PPOBLG and catalyst (B) and the PPOBLG (containing a water impurity, (C)). This figure shows that the signals of PPOBLG are not significantly shifted by the presence of the catalyst.

7. Extrapolation of the effective enhancement related to the thermal signal

To estimate an enhancement factor $\mathcal{E}$ for those concentrations where no thermal signal is available for calculation, we plotted the results of the intensity of the thermal signals of the series of concentrations and fitted the data with a second order polynomial. The obtained equation ($y = 0.4316x^2 + 0.4469x + 0.0004$) is used to estimate the intensity of thermal signals at the low concentrations of 0.071 and 0.053 μM. Below those concentrations no thermal signals could be determined.

8. Reaction progress during hydrogenation

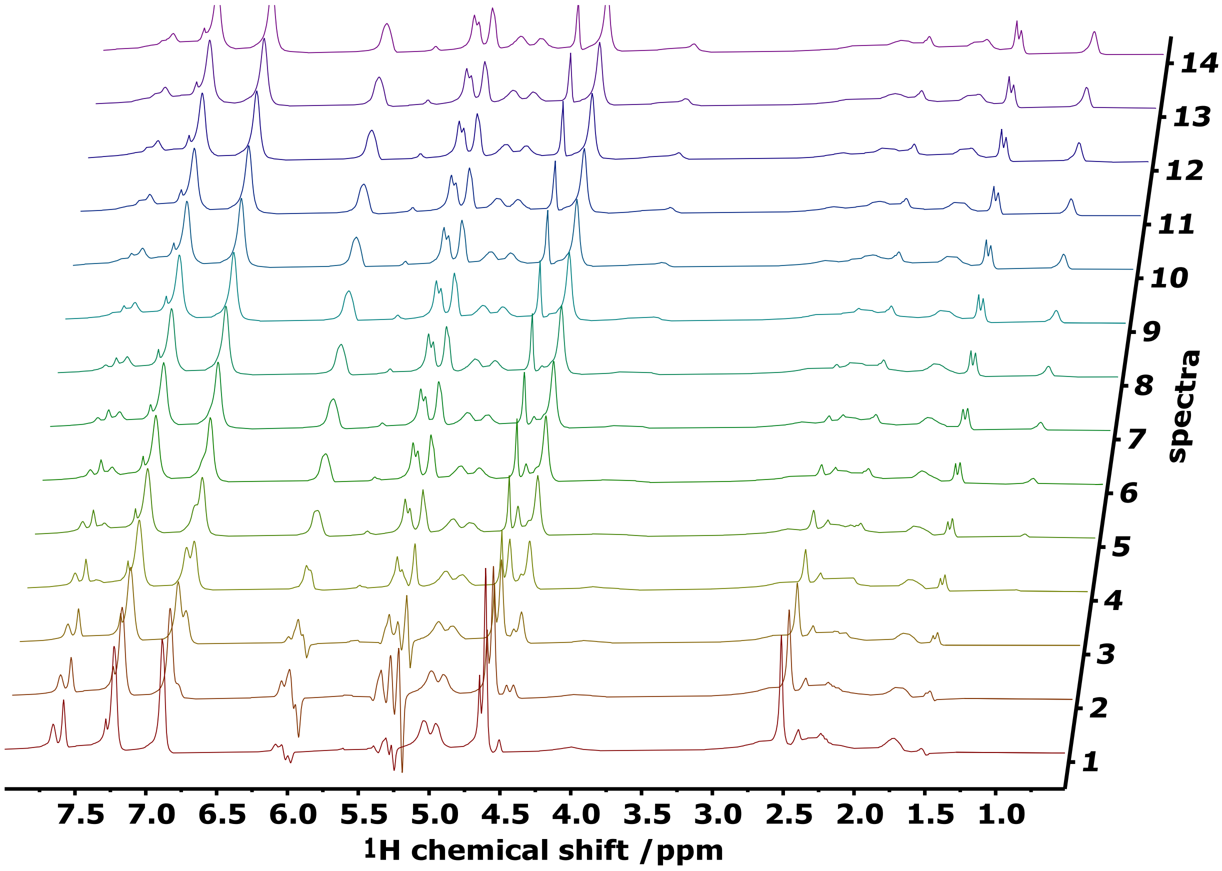

Figure 6: Control of the reaction progress during the hydrogenation, in order to observe the change of the signals over time. This also allowed to determine the end point of the reaction with 16 mg PPOBLG, which is then precipitated. Due to the presence of the catalyst, the signals of the newly formed allyl and alkyl moiety may be shifted compared to the assignment of the precipitated products. Between single ^{1}H experiments in the pseudo-2D experiments a hydrogenation time of 10 s was used. There is no additional delay between the acquisition of the spectra. (500MHz, 298K, in $CDCl_3$).

References

1. Bowers, C. R. & Weitekamp, D. P. Parahydrogen and synthesis allow dramatically enhanced nuclear alignment. *J. Am. Chem. Soc.* **109,** 5541–5542; 10.1021/ja00252a049 (1987).
2. Kiryutin, A. S. *et al.* A highly versatile automatized setup for quantitative measurements of PHIP enhancements. *J. Magn. Res.* **285,** 26–36; 10.1016/j.jmr.2017.10.007 (2017).
